# Long-term Effects of Concomitant Lateral Meniscal Management on ACL Reconstruction Revision Rate and Secondary Meniscal and Cartilaginous Injuries

**DOI:** 10.1177/23259671251330655

**Published:** 2025-05-05

**Authors:** Jonas Olsson Wållgren, Jacob F. Oeding, Janina Kaarre, Eric Hamrin Senorski, Volker Musahl, Kristian Samuelsson

**Affiliations:** † Department of Orthopaedics, Institute of Clinical Sciences, Sahlgrenska Academy, University of Gothenburg, Gothenburg, Sweden; ‡ Sahlgrenska Sports Medicine Center, Gothenburg, Sweden; § Department of Orthopaedics, NU Hospital Group, Trollhättan, Sweden; ‖ School of Medicine, Mayo Clinic Alix School of Medicine, Rochester, Minnesota, USA; ¶ Unit of Physiotherapy, Department of Health and Rehabilitation, Institute of Neuroscience and Physiology, Sahlgrenska Academy, University of Gothenburg, Gothenburg, Sweden; # Department of Orthopaedic Surgery, UPMC Freddie Fu Sports Medicine Center, University of Pittsburgh Medical Center, Pittsburgh, Pennsylvania, USA; ** Department of Orthopaedics, Sahlgrenska University Hospital, Mölndal, Sweden; Investigation performed at Department of Orthopaedics, Institute of Clinical Sciences, Sahlgrenska Academy, University of Gothenburg, Gothenburg, Sweden

**Keywords:** meniscus, repair, resection, ACL, anterior cruciate ligament, reconstruction, revision, cartilage

## Abstract

**Background:**

When injuring the anterior cruciate ligament (ACL), simultaneous meniscal tears often occur. However, little data describe ACL reconstruction (ACLR) and the subsequent risk of revision ACLR, as well as cartilaginous and meniscal damage after primary ACLR with different treatments for injury of the lateral meniscus (LM).

**Purpose:**

To investigate (1) how different LM treatments during primary ACLR affect the odds of revision ACLR and the cartilaginous and meniscal status at revision in a long-term follow-up and (2) whether the increased risk of subsequent meniscal and cartilaginous injuries seen with meniscal resection in the short term would continue in the long term, and (3) the increased risk with subsequent meniscal injuries with meniscal repair.

**Study Design:**

Cohort study; Level of evidence, 3.

**Methods:**

Data from the Swedish National Knee Ligament Registry were used. The study compared 2 groups: patients with isolated ACLR and patients with ACLR and concomitant LM injury, further subdividing the latter into 4 treatment types: repair, resection, both, and left in situ. Patients with medial meniscal injuries were excluded. The main outcomes studied were the odds for revision ACLR and the cartilaginous and meniscal status at revision 5 and 10 years after primary surgery.

**Results:**

Data for 5 years were available for 22,208 patients and data for 10 years were available for 11,058 patients. Compared with isolated ACLR, patients with concomitant LM injury left in situ had higher odds of revision ACLR at both 5 years (odds ratio [OR], 1.49; 95% CI, 1.14-1.95; *P* = .004) and 10 years (OR, 1.55; 95% CI, 1.09-2.19; *P* = .01), while those with LM repair had higher odds at 5 years (OR, 1.73; 95% CI, 1.23-2.43; *P* = .002). LM repair also increased the odds of subsequent meniscal injuries at 5 years (OR, 3.52; 95% CI, 1.76-7.03; *P* = .0004) and 10 years (OR, 7.26; 95% CI, 1.67-31.52; *P* = .008). Partial meniscectomy did not show an increased risk of cartilage injury, subsequent meniscal injury, or revision ACLR at long-term follow-up compared with the control group.

**Conclusion:**

This demonstrates that over 5 and 10 years, LM repair during primary ACLR is associated with an increased risk of revision ACLR and subsequent meniscal injuries. The higher risk of revision ACLR when the LM is left in situ emphasizes the need for careful consideration of treatment methods. Conversely, partial meniscectomy did not show an associated increased risk of cartilaginous injury in the long term and is associated with a lower risk of subsequent meniscal injury and revision ACLR than other treatment options, raising questions about whether preserving the meniscus at all costs is always the best approach.

Rupture of the anterior cruciate ligament (ACL) is a devastating injury that frequently results from a sudden pivoting or cutting maneuver during sporting activity. For cases resulting in instability, arthroscopic ACL reconstruction (ACLR) is the gold standard treatment to restore knee stability and reduce the risk of subsequent meniscal and cartilaginous injury.^6,7^ A variety of factors have been identified as influencing the risk for ACLR failure, including patient-specific characteristics such as femoral notch size, activity level, and knee malignment, as well as other surgery-specific features such as graft type and timing of surgery.^3,10,24^ Given that ACLR failure is associated with an increased risk for subsequent damage to menisci and knee cartilage, including substantial increases in the risk for posttraumatic osteoarthritis,^4,12,18^ identifying strategies to reduce the risk for ACLR failure is critical.

Importantly, ACL injuries commonly present with simultaneous tearing of the meniscus, with the lateral meniscus (LM) more commonly injured than the medial meniscus in acute settings.^14^ There are different treatment options for the injured meniscus during ACLR. It may be repaired, resected, or left in situ, depending on the type of meniscal tear and patient characteristics.^23^ Repair is increasingly preferred because of evidence suggesting improved quality of life and decreased risk for posttraumatic osteoarthritis when compared with partial meniscal resection.^15,21^ Interestingly, though, while LM deficiency has been linked to persistent knee laxity,^9^ no difference in revision ACLR rates between ACL-injured knees with the meniscus repaired, resected, or left in situ has been reported at 2-year follow-up.^19^ However, when compared with patients undergoing isolated primary ACLR, higher odds of meniscal and cartilaginous injuries with LM resection and higher odds of meniscal injury with LM repair have been reported at the time of revision ACLR within 2 years of primary ACLR.^19^ Nevertheless, how different management strategies for concomitant LM tears in the setting of primary ACLR compare in the long term remains unknown.

The purpose of this study was to determine how the management strategy for a concomitant LM injury—either repaired, resected, or left in situ during primary ACLR—affects the odds for revision ACLR. A second purpose was to evaluate cartilaginous and meniscal status at long-term follow-up, at the time of revision within 5 and 10 years of primary ACLR. We hypothesized that the differences observed in the short term would possibly continue in the long term, with higher odds of meniscal and cartilaginous injuries at the time of revision ACLR within 5 and 10 years of primary ACLR with LM resection as well as higher odds of meniscal injury at the time of revision ACLR within 5 and 10 years of primary ACLR with LM repair, when compared with those of isolated primary ACLR.

## Methods

This study was approved by the Swedish Ethical Review Authority, and it was conducted according to the Strengthening the Reporting of Observational Studies in Epidemiology guidelines.^25^

The study design has previously been described in detail in another publication from our research team.^19^ In summary, the included data were extracted in 2022 from the Swedish National Knee Ligament Registry (SNKLR), which is a registry, developed in 2005, that includes 90% of the patients undergoing primary ACLR in Sweden and consists of data from both the surgeon and the patient. The patients give consent to participate in research when they enter the registry. The registry has been more clearly described in previous literature.^1,2,20,22^

### Data Collection and Study Sample

Data regarding patients undergoing primary ACLR between 2005 and 2018 were collected from the SNKLR. Patients with (1) 5 years of follow-up data and (2) 10 years of follow-up data separately were included in this study. Exclusion criteria were age <15 years at the time of surgery; any previous knee surgery; concomitant fracture, medial meniscal injury, or concomitant posterior cruciate ligament or neurovascular injury; and those undergoing allograft, synthetic graft, double-bundle ACLR, or surgical treatment for concomitant medial collateral ligament or lateral collateral ligament injury.

First, 2 different groups were formed from the included patients: (1) unexposed comparison group of patients undergoing isolated ACLR without concomitant LM injury and (2) exposed group of patients undergoing ACLR with concomitant injury to the LM. Furthermore, the exposed group was divided into 4 subgroups depending on the treatment of the LM injury received: (1) meniscal repair, (2) meniscal resection, (3) both repair and resection, and (4) left in situ (nonoperative management). Additionally, information regarding the patient (sex, age, and body mass index) and surgical (graft type and time from injury to surgery) characteristics was also extracted from the SNKLR. However, no data were available regarding the specific tear type, size, or location in the menisci or number of meniscal sutures or devices used if it repair was carried out.

### Outcome Measures

The main outcome of interest was the rate of ipsilateral ACL revision when comparing different meniscal treatments at 5 years and 10 years after primary ACLR, with simultaneous LM injury. The secondary outcome of interest was if there were any new simultaneous meniscal or cartilaginous injuries at the time of revision ACLR, at 5 years, or at 10 years, respectively.

### Statistical Analysis

The statistical analyses were performed using SAS System for Windows software (Version 9.4; SAS Institute). All significance tests were conducted at the 5% significance level. Count and proportion were used to present categorical variables, while mean with standard deviation and median with minimum and maximum were used for presenting continuous and ordinal data, respectively. Univariable logistic regression analysis was used both to determine whether different treatments received on the concomitant LM injury during primary ACLR affected the odds of ACL revision and to determine cartilaginous or meniscal status at the time of revision ACLR; results were presented as odds ratio (OR), 95% CI, and *P* value. Moreover, the area under the receiver operating characteristic curve (AUC) was calculated with a 95% CI. The AUC varied between 0.5 and 1.00, where an AUC between 0.9 and 1.00 represented excellent predictive capacity and between 0.5 and 0.7 indicated poor predictive capacity of the statistical model.^11^

## Results

### Baseline Characteristics

**Figure 1.**
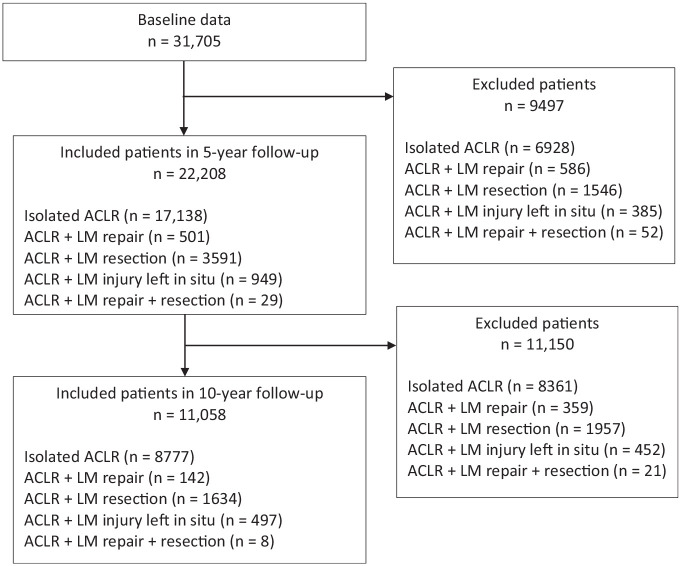
Study flowchart. ACLR, anterior cruciate ligament reconstruction; LM, lateral meniscus.

During this study period, a total of 31,705 primary ACLRs with available baseline data were registered in the SNKLR (Figure 1). Among the included patients, as seen in Table 1, the mean ± SD age at time of surgery was 27.1 ± 9.9 years, 56.1% were male, and the mean body mass index was 24.5 ± 3.3 kg/m^2^. The time from injury to surgery was 17.0 ± 30.8 months. Furthermore, the mean age of the included patients was slightly higher in the control group than the LM treatment groups (meniscal repair, partial resection, both repair and resection, and nonoperative management). Additionally, there were slightly lower numbers of patients involved in pivoting sports activity at the time of injury, also shown in detail in Table 1.

**Table 1:** Baseline Characteristics^*a*^

	Total	Isolated ACLR	ACLR + LM Repair	ACLR + LM Resection	ACLR + LM Injury Left In Situ	ACLR + LM Repair + LM Resection
	(N = 31,705)	(n = 24,066)	(n = 1087)	(n = 5137)	(n = 1334)	(n = 81)
Age at time of injury, y	25.6 (9.6)	26.0 (9.8)	22.5 (8.3)	24.7 (8.7)	24.0 (8.5)	21.4 (7.3)
	23 (1-70)	23 (1-70)	20 (1-57)	22 (7-64)	21 (11-58)	19 (11-50)
Age at time of surgery, y	27.1 (9.9)	27.6 (10.1)	23.8 (8.6)	26.2 (9.1)	25.2 (8.8)	22.5 (7.4)
	25 (15-71)	25 (15-71)	21 (15-58)	24 (15-66)	23 (15-60)	20 (15; 50)
Male sex	17,794 (56.1)	12,887 (53.5)	617 (56.8)	3480 (67.7)	751 (56.3)	59 (72.8)
BMI, kg/m^2^	24.5 (3.3)	24.5 (3.3)	23.8 (2.8)	24.8 (3.2)	24.4 (3.3)	23.9 (3.0)
	24.2 (15.4-49.8)	24.1 (15.4-49.8)	23.4 (17.4-35.4)	24.3 (16-42.7)	23.9 (17.8-44.1)	23.4 (19-29.6)
Smoking (yes)^*b*^	784 (5.2)	605 (4.8)	26 (4.3)	117 (4.3)	34 (4.9)	2 (4.3)
Injury						
Right knee (no variable found)						
Time from injury to surgery, mo	17.0 (30.8)	17.4 (31.3)	13.5 (27.0)	16.9 (29.7)	14.7 (27.3)	10.5 (19.7)
	7.5 (0-458.4)	7.7 (0-458.4)	5.9 (0.1-247.9)	7.2 (0.1-434.9)	7.2 (0.1-358.9)	5.4 (0.8-140.8)
Concomitant injury (except LM)	7917 (25.0)	5499 (22.8)	326 (30.0)	1663 (32.4)	386 (28.9)	43 (53.1)
Cartilaginous injury (yes)						
Lateral femoral condyle	1570 (5.0)	837 (3.5)	85 (7.8)	540 (10.5)	94 (7.0)	14 (17.3)
Medial femoral condyle	4218 (13.3)	3083 (12.8)	164 (15.1)	787 (15.3)	162 (12.1)	22 (27.2)
Lateral patella	697 (2.2)	489 (2.0)	32 (2.9)	145 (2.8)	28 (2.1)	3 (3.7)
Medial patella	1276 (4.0)	934 (3.9)	36 (3.3)	223 (4.3)	67 (5.0)	7 (8.6)
Lateral tibial plateau	1754 (5.5)	1026 (4.3)	90 (8.3)	511 (9.9)	112 (8.4)	15 (18.5)
Medial tibial plateau	1058 (3.3)	810 (3.4)	26 (2.4)	193 (3.8)	27 (2.0)	3 (3.7)
Trochlea	743 (2.3)	518 (2.2)	37 (3.4)	156 (3.0)	28 (2.1)	4 (4.9)
Collateral ligament injury (yes)						
LCL	249 (0.8)	159 (0.7)	15 (1.4)	55 (1.1)	19 (1.4)	1 (1.2)
MCL	1189 (3.8)	813 (3.4)	67 (6.2)	212 (4.1)	89 (6.7)	8 (9.9)
PLC injury (yes)	23 (0.1)	16 (0.1)	1 (0.1)	6 (0.1)	0	0
ACL graft (yes)						
Patellar tendon autograft	1848 (5.8)	1407 (5.8)	60 (5.5)	310 (6.0)	67 (5.0)	4 (4.9)
Semitendinosus autograft	28,977 (91.4)	22,022 (91.5)	966 (89.0)	4688 (91.3)	1238 (92.8)	63 (77.8)
Quadriceps tendon autograft	525 (1.7)	355 (1.5)	49 (4.5)	89 (1.7)	18 (1.3)	14 (17.3)
Activity at time of injury (yes)						
Alpine skiing	4719 (14.9)	3830 (15.9)	156 (14.4)	541 (10.5)	182 (13.6)	10 (12.3)
Pivoting sport^*c*^	21,425 (67.6)	15,881 (66.0)	780 (71.8)	3748 (73.0)	953 (71.4)	63 (77.8)
Nonpivoting sport^*d*^	1301 (4.1)	956 (4.0)	44 (4.0)	234 (4.6)	65 (4.9)	2 (2.5)
Other physical activity^*e*^	1165 (3.7)	915 (3.8)	29 (2.7)	173 (3.4)	46 (3.4)	2 (2.5)
Traffic related	490 (1.5)	364 (1.5)	20 (1.8)	85 (1.7)	19 (1.4)	2 (2.5)
Other	2544 (8.0)	2064 (8.6)	57 (5.2)	353 (6.9)	68 (5.1)	2 (2.5)

a Values are presented as n (%) for categorical variables and mean ± SD and median (minimum-maximum) for continuous and ordinal variables, respectively. The sums may vary because of missing values. The variables with missing values (n [%] of the total sample) were age at time of injury (712 [2.2]), BMI (15,213 [48.0]), smoking (16,754 [52.8]), time from injury to surgery (751 [2.4]), ACL graft (355 [1.1]), and activity at time of injury (61 [0.2]). ACL, anterior cruciate ligament; ACLR, anterior cruciate ligament reconstruction; BMI, body mass index; LCL, lateral collateral ligament; LM, lateral meniscus; MCL, medial collateral ligament; PLC, posterior lateral corner.

b The numbers are calculated including missing values.

c Pivoting sports included American football/rugby, basketball, dancing, floorball, gymnastics, handball, ice hockey/bandy, martial arts, racket sports, soccer, volleyball, and wrestling.

d Nonpivoting sports included cross-country skiing, cycling, horseback riding, motocross/endure, skateboarding, snowboarding, and surfing/wakeboarding.

e Other physical activity included recreational sports, exercise, and trampoline.

### Revision ACLR Rate at 5 and 10 Years by LM Treatment at Primary ACLR

**Figure 2.**
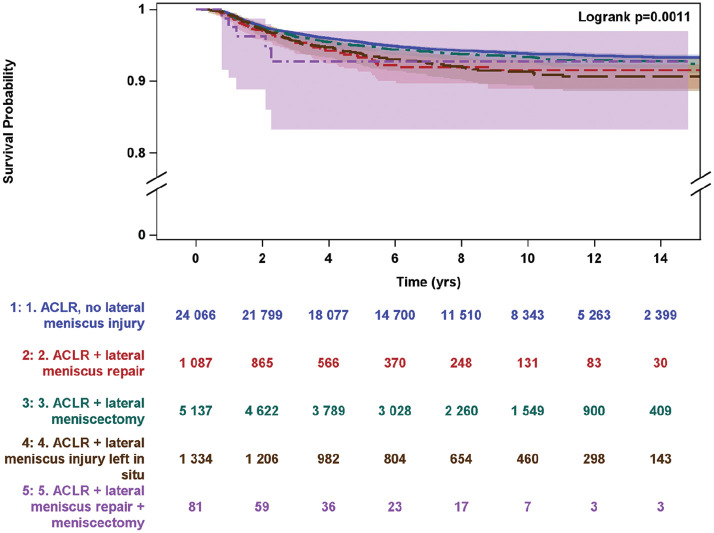
Survival analysis by lateral meniscal status at primary ACL comparing isolated anterior cruciate ligament reconstruction (ACLR), ACLR with concomitant lateral meniscal (LM) repair (ACLR + LM repair), ACLR with concomitant LM resection (ACLR + LM resection), ACLR with concomitant LM injury left in situ (ACLR + LM injury left in situ), and ACLR with concomitant LM repair and resection (ACLR + LM repair + LM resection).

Five-year follow-up data were available for 22,208 patients and 10-year follow-up data were available for 11,058 patients (Figure 1). In total, 1031 of the included patients underwent revision ACLR within 5 years after primary ACLR, and 568 patients underwent revision ACLR within 10 years. Compared with those who underwent isolated ACLR, patients with concomitant LM injury left in situ at primary ACLR had higher odds of revision ACLR, both at 5 years (OR, 1.49; 95% CI, 1.14-1.95; *P* = .004) and at 10 years (OR, 1.55; 95% CI, 1.09-2.19; *P* = .01). Compared with those who underwent isolated ACLR, patients treated with LM repair had higher odds of revision ACLR in 5 years (OR, 1.73; 95% CI, 1.23-2.43; *P* = .002), as seen in Table 2. A survival analysis comparing isolated ACLR, ACLR with concomitant LM repair, ACLR with concomitant LM resection, and ACLR with concomitant LM injury left in situ is provided in Figure 2.

**Table 2:** Revision ACLR Rate by LM Status at Primary ACLR for 5 and 10 Years^*a*^

	n	n Missing	ACLR, no LM injury vs different LM treatment groups	n (%) of Event	OR (95% CI) of Revision ≤5 Years	*P*	AUC (95% CI)
5 years n=22,208	17,138	0	ACLR, no LM injury vs ACLR, no LM injury	756 (4.4)	1.00	.001^*b*^	
	501	0	vs ACLR + LM repair	37 (7.4)	1.73 (1.23-2.43)	.002	
	3591	0	vs ACLR + lateral meniscectomy	175 (4.9)	1.11 (0.94-1.31)	.22	
	949	0	vs ACLR + LM injury left in situ	61 (6.4)	1.49 (1.14-1.95)	.004	
	29	0	vs ACLR + LM repair + meniscectomy	2 (6.9)	1.61 (0.38-6.76)	.52	0.52 (0.51-0.54)
10 years n=11,058	8777	0	ACLR, no LM injury vs ACLR, no LM injury	434 (4.9)	1.00	.07^*b*^	
	142		vs ACLR + LM repair	11 (7.7)	1.61 (0.87-3.01)	.13	
	1634		vs ACLR + lateral meniscectomy	85 (5.2)	1.05 (0.83-1.34)	.66	
	497		vs ACLR + LM injury left in situ	37 (7.4)	1.55 (1.09-2.19)	.01	
	8		vs ACLR + LM repair + meniscectomy	1 (12.5)	2.75 (0.34-22.38)	.34	0.52 (0.50-0.54)

a All tests were performed with univariable logistic regression. *P* values, OR, and area under ROC curve are based on original values and not on stratified groups. ACLR, anterior cruciate ligament reconstruction; AUC, area under the receiver operating characteristic curve; LM, lateral meniscus; OR, odds ratio.

b *P* value for the entire effect/factor/variable.

### Concomitant Injury at 5 and 10 Years by LM Treatment Status at Primary ACLR

Five-year follow-up data on revision ACLR patients were available in 1031 patients regarding concomitant meniscal or cartilaginous injuries in total from all subgroups of LM treatment at primary ACLR. At 10 years, follow-up data were available for 568 patients.

Patients with LM repair at primary ACLR had higher odds (OR, 3.52; 95% CI, 1.76-7.03; *P* = .0004) of concomitant meniscal injuries (medial or lateral) at the time of revision 5 years after ACLR than patients undergoing isolated primary ACLR, as shown in Table 3. At 10 years, patients with LM repair at primary ACLR likewise had higher odds (OR, 7.26; 95% CI, 1.67-31.52; *P* = .008) of concomitant meniscal injuries. No other treatment to the menisci at primary ACLR showed any increased odds of concomitant meniscal injury at 5 or 10 years. Additionally, regarding concomitant cartilaginous injury at time of revision ACLR, no increase in odds were found in any LM treatment group compared with patients undergoing isolated primary ACLR, at time of revision either at 5 years or 10 years follow-up (Table 4).

**Table 3:** Concomitant Meniscal Injuries at Revision ACLR by LM Status at Primary ACLR for 5 and 10 Years^*a*^

	n	n Missing	ACLR no LM injury vs different LM treatment groups	n (%) of Meniscal injuries	OR (95% CI)^*b*^	*P*	AUC (95% CI)
5 years n=1031	756	0	ACLR, no LM injury vs ACLR, no LM injury	260 (34.4)	1.00	.007^*c*^	
	37	0	vs ACLR + LM repair	24 (64.9)	3.52 (1.76-7.03)	.0004	
	175	0	vs ACLR + lateral meniscectomy	70 (40.0)	1.27 (0.91-1.78)	.16	
	61	0	vs ACLR + LM injury left in situ	23 (37.7)	1.15 (0.67-1.98)	.60	
	2	0	vs ACLR + LM repair + meniscectomy	1 (50.0)	1.91 (0.12-30.62)	.65	0.54 (0.51-0.57)
10 years n=568	434	0	ACLR, no LM injury vs ACLR, no LM injury	149 (34.3)	1.00	.09^*c*^	
	11		vs ACLR + LM repair	9 (81.8)	7.26 (1.67-31.52)^*d*^	.008^*d*^	
	85		vs ACLR + lateral meniscectomy	33 (38.8)	1.22 (0.75-1.97)^*d*^	.42^*d*^	
	37		vs ACLR + LM injury left in situ	11 (29.7)	0.83 (0.40-1.72)^*d*^	.61^*d*^	
	1		vs ACLR + LM repair + meniscectomy	0 (0.0)	0.57 (0.01-60.27)^*d*^	.81^*d*^	0.54 (0.50-0.58)

a All tests are performed with univariable logistic regression. *P* values, OR, and area under ROC curve are based on original values and not on stratified groups. ACLR, anterior cruciate ligament reconstruction; AUC, area under the receiver operating characteristic curve; LM, lateral meniscus; OR, odds ratio.

b (7) Meniscal injury (medial and/or lateral) at 1st revision ACLR.

c *P* value for the entire effect/factor/variable.

d Firth penalized maximum likelihood estimation to reduce bias in the parameter estimates. Firth D. Bias reduction of maximum likelihood estimates. *Biometrika*. 1993;80:27-38. Heinze G, Schemper M. A solution to the problem of separation in logistic regression. *Stat Med*. 2002;21:2409-2419.

**Table 4:** Concomitant Cartilaginous Injuries at Revision ACLR by LM Status at Primary ACLR^*a*^

	n	n Missing	ACLR no LM injury vs different LM treatment groups	n (%) of Cartilaginous injuries	OR (95% CI)^*b*^	*P*	AUC (95% CI)
5 years n=1031	756	0	ACLR, no LM injury vs ACLR, no LM injury	234 (31.0)	1.00	.58^*c*^	
	37	0	vs ACLR + LM repair	15 (40.5)	1.52 (0.78-2.98)	.22	
	175	0	vs ACLR + lateral meniscectomy	62 (35.4)	1.22 (0.87-1.73)	.25	
	61	0	vs ACLR + LM injury left in situ	19 (31.1)	1.01 (0.57-1.77)	.97	
	2	0	vs ACLR + LM repair + meniscectomy	1 (50.0)	2.23 (0.14-35.82)	.57	0.52 (0.49-0.55)
10 years n=568	434	0	ACLR, no LM injury vs ACLR, no LM injury	161 (37.1)	1.00	.86^*c*^	
	11	0	vs ACLR + LM repair	5 (45.5)	1.43 (0.43-4.77)^*d*^	.56^*d*^	
	85	0	vs ACLR + lateral meniscectomy	36 (42.4)	1.25 (0.78-2.00)^*d*^	.36^*d*^	
	37	0	vs ACLR + LM injury left in situ	13 (35.1)	0.93 (0.46-1.88)^*d*^	.85^*d*^	
	1	0	vs ACLR + LM repair + meniscectomy	0 (0.0)	0.51 (0.00-53.49)^*d*^	.77^*d*^	0.52 (0.48-0.56)

a All tests are performed with univariable logistic regression. *P* values, OR, and area under ROC curve are based on original values and not on stratified groups. ACLR, anterior cruciate ligament reconstruction; AUC, area under the receiver operating characteristic curve; LM, lateral meniscus; OR, odds ratio.

b (7) Cartilaginous injury at 1st revision ACLR

c P value for the entire effect/factor/variable.

d Firth penalized maximum likelihood estimation to reduce bias in the parameter estimates. Firth D. Bias reduction of maximum likelihood estimates. *Biometrika*. 1993;80:27-38. Heinze G, Schemper M. A solution to the problem of separation in logistic regression. *Stat Med*. 2002;21:2409-2419.

## Discussion

The primary findings of this study include the following: (1) when compared with patients undergoing isolated primary ACLR without concomitant LM injury, patients with concomitant LM injury left in situ at primary ACLR experienced increased odds of revision ACLR, both at 5 years and at 10 years after primary ACLR; (2) patients with concomitant LM injury, regardless of the treatment received for the LM injury, experienced no difference in odds of cartilaginous injury at 5 and 10 years postoperatively compared with those without concomitant LM injury who underwent isolated primary ACLR; and (3) patients who underwent LM repair at the time of primary ACLR experienced an increased risk for both revision ACLR at 5 years and meniscal injury at the time of revision ACLR at 5 years and 10 years, while patients who underwent LM resection at the time of primary ACLR experienced neither an increased risk of revision ACLR nor meniscal or cartilaginous injury at the time of revision ACLR 5 or 10 years after primary ACLR.

Relative to that of patients undergoing isolated primary ACLR, a higher risk of revision ACLR at 5 and 10 years was observed when the LM injury was left in situ. While cadaveric studies have shown that LM lesions in the setting of ACL-deficient knees significantly increase rotation and translation under a coupled valgus stress and internal rotation torque/pivot-shift test,^9^ an increase in risk for revision ACLR due to this increase in knee laxity has not been reported in clinical studies.^13,17,19^ In fact, when comparing the rate of revision ACLR at 2-year follow-up between patients who underwent isolated primary ACLR, ACLR with concomitant LM repair, ACLR with concomitant LM resection, and ACLR with the LM injury left in situ, Persson et al^19^ found no difference in the risk for revision ACLR. Previous literature has also reported no difference in revision rates between patients undergoing isolated ACLR and patients with concomitant meniscal injuries in the short term.^13,17^ The present study, however, can report an increased risk for revision ACLR in patients with the LM left in situ at long-term follow-up. This may be explained in the context of the crucial role of the LM as a stabilizer in the knee joint and the fact that patients with the LM left in situ during primary ACLR may be more prone to experiencing progression of the tear left in situ and degeneration with loss of function of the LM over time. It is possible that this loss of function occurs over the long term in such a way that any effects on knee stability are not observed in the short term but begin to put patients at increased risk for subsequent retears at 5 and 10 years after primary ACLR. It is possible that this is because surgeons may not be using appropriate indications for in situ treatment.

The present study did not find an association between primary ACLR, with or without concomitant LM injury and/or management, and risk for cartilaginous injury at the time of revision ACLR 5 or 10 years after primary ACLR. This finding contrasts with the results seen at 2 years after primary ACLR, which found an increased risk for cartilaginous injury in patients who underwent concomitant LM resection compared with patients who underwent isolated primary ACLR.^19^ It is possible that LM resection at the time of ACLR results in a loss of meniscal function that prematurely places increased stress on articular cartilage and predisposes patients to cartilaginous injury faster than patients with isolated primary ACLR or concomitant LM repair. This suggests that repair of the LM may be chondroprotective in the short term, but in the long term, all patients with primary ACL tears ultimately experience the same risk for cartilaginous injury at the time of revision ACLR.

Finally, we found that patients who underwent LM repair at the time of primary ACLR experienced an increased risk for both revision ACLR at 5 years and meniscal injury at the time of revision ACLR at 5 years and 10 years, while patients who underwent LM resection at the time of primary ACLR experienced neither an increased risk of revision ACLR nor meniscal or cartilaginous injury at the time of revision ACLR 5 or 10 years after primary ACLR. While the finding that those with LM repair experience an increased risk for meniscal injury at 5 years is consistent with the results seen at 2 years, the increased risk for revision ACLR at 5 and 10 years with LM repair as well as the lack of an increased risk for meniscal and cartilaginous injuries with LM resection differ from the results observed by Persson et al^19^ at 2 years. While there are strong incentives to prioritize meniscus-sparing treatment strategies—with damage to the meniscus associated with loss of the hoop stress mechanism and a dose-dependent relation between the volume of meniscus lost and progression of degenerative changes within the knee^16^—successful meniscal repair relies on careful consideration of multiple factors, including tear location and the resulting blood supply.^5,8^ Surgeons may have different criteria for when to choose repair and when to choose meniscectomy. They may attempt meniscal repair in less suitable injuries in younger, more active individuals, or large tears, and the possible failure of meniscal repair may result in increased stress on the ACL graft, leading to future need for revision. Also, long wait times for surgery could have an effect if the LM tears, in some cases, could be presumed to be chronic and have a lower healing capacity if a meniscal repair were performed. Thus, in the context of the equal risk for subsequent meniscal injury at the time of revision ACLR observed between ACLR with concomitant LM resection and isolated ACLR, these results suggest that more attention is warranted in identifying concomitant meniscal tears that may be suitable for repair in the setting of primary ACLR. This is particularly salient when noting the increased risk for revision ACLR observed in patients who underwent primary ACLR with LM repair versus LM resection. Patients with tear patterns not suitable for repair may be more prone to experience subsequent meniscal injury (medial or lateral) and ACLR failure if a concomitant LM repair is performed. Of course, given the relatively recent focus on preserving the meniscus as well as advances in repair techniques, it is also possible that long-term outcomes of ACLR with concomitant LM repair will improve over time, particularly as more high-level, comparative studies investigating the optimal meniscal repair technique, as well as rehabilitation protocol, for different tear patterns, locations, and ACL reconstruction techniques become available.

### Limitations

This study has limitations that warrant discussion. First, despite its large sample size, the study is unable to determine a causal relationship between LM management and outcomes of ACLR because of its registry design. Only univariate analyses were performed in the study. Second, it is possible that this study underestimates the rate of primary ACLR failure, cartilaginous injuries, and meniscal injuries within 5 and 10 years of primary ACLR, given that the study only included cases that underwent surgical management for primary ACLR failure and did not detect future meniscal surgery without the need for revision ACL surgery. The fact that only those cases were included also gives no possibility of comparing subsequent injuries or development of knee osteoarthritis with those who did not undergo revision ACLR. Furthermore, data on the specific tear pattern, size, and location of the LM tear, preoperative laxity, and reasons for a specific meniscal treatment being chosen were not available. This prevents additional analysis of potential associations between tear type, specific surgical repair method, and outcomes after primary ACLR and may also lead to possible confounding by the failure to state why some of the meniscal injuries were resected, repaired, or treated nonoperatively. Finally, despite the fact that it is an optimal study population for a long-term follow-up, some of the subgroups with different treatment options were small relative to the study population.

## Conclusion

This long-term follow-up regarding how different LM treatments during primary ACLR affect the odds of ACL revision and findings of cartilaginous and meniscal injury at revision suggest that over 5 and 10 years, meniscal repair during ACLR is associated with an increased odds of revision and concomitant meniscal injury, raising doubts whether meniscal repair provides sustained benefits over time. Clinicians should be aware of the increased revision ACL rate when considering LM repair. The higher odds of revision when the LM injury is left in situ underscores the critical role of the meniscus as a stabilizer of the knee joint, emphasizing the need for careful consideration of treatment methods to optimize long-term knee stability and function. Conversely, partial meniscectomy did not increase the odds of cartilaginous injury at 5 or 10 years and is associated with a lower risk of further meniscal injury and ACL revision. These findings do not state that the partial meniscectomy is always beneficial for patients, but they raise questions about whether preserving the meniscus at all costs is always the best approach.
